# Inhibition of Biofilm Formation by the Synergistic Action of EGCG-S and Antibiotics

**DOI:** 10.3390/antibiotics10020102

**Published:** 2021-01-21

**Authors:** Shrameeta Shinde, Lee H. Lee, Tinchun Chu

**Affiliations:** 1 Department of Biology, Montclair State University, Montclair, NJ 07043, USA; shrameeta@gmail.com; 2 Department of Microbiology, Miami University, Oxford, OH 45056, USA; 3 Department of Biological Sciences, Seton Hall University, South Orange, NJ 07079, USA

**Keywords:** biofilm, green tea polyphenol, antibiotics, EGCG-S

## Abstract

Biofilm, a stress-induced physiological state, is an established means of antimicrobial tolerance. A perpetual increase in multidrug resistant (MDR) infections associated with high mortality and morbidity have been observed in healthcare settings. Multiple studies have indicated that the use of natural products can prevent bacterial growth. Recent studies in the field have identified that epigallocatechin gallate (EGCG), a green tea polyphenol, could disrupt bacterial biofilms. A modified lipid-soluble EGCG, epigallocatechin-3-gallate-stearate (EGCG-S), has enhanced the beneficial properties of green tea. This study focuses on utilizing EGCG-S as a novel synergistic agent with antibiotics to prevent or control biofilm. Different formulations of EGCG-S and selected antibiotics were used to study their combinatorial effects on biofilms produced by five potential pathogenic bacteria, *Escherichia coli*, *Pseudomonas aeruginosa*, *Staphylococcus aureus*, *Staphylococcus* *epidermidis*, and *Mycobacterium smegmatis*. The crystal violet (CV) assay and the sensitive fluorescence-based resazurin biofilm viability assay were used to assess the biofilm production. Our results identified optimal formulation for each bacterium, effectively inhibiting biofilm formation to an extent of 95–99%. Colony-forming unit (CFU) and cell viability analyses showed a decrease of viable bacteria. These results depict the potential of EGCG-S as a synergistic agent with antibiotics and as an anti-biofilm agent.

## 1. Introduction

Selective environmental pressures force bacteria to adapt by altering their growth state. One preferred state is biofilm, which exists in almost 90% of bacteria. Biofilm is a three-dimensional, multicellular surface-tethered bacterial aggregation embedded in an extracellular matrix (ECM). During biofilm formation, planktonic cells attach to surface and transition to the sessile state to secrete an extracellular polymeric substance (EPS) forming a protective barrier against abiotic and biotic stressors. Upon maturation of biofilm, the cells are shed for dispersal which then transition into planktonic cells [1]. Biofilm is an inherent physiological and regulatory strategy for being refractory to antimicrobial treatments and the host immune system [1,2]. Clinically relevant biofilm-associated infections are either tissue or device-related infections. Chronic tissue infections include wounds, dental plaque, urinary tract infection, cystic fibrosis, and so on, while medical devices like catheters, prosthetic heart valves, orthopedic implants, and so on are colonized by bacteria [3].

Up to 65% and 80% of microbial and chronic infections respectively are linked to bacterial biofilms [4]. Additionally, recurrent bacterial infections are due to the persistent nature of the biofilms [1]. Currently, ESKAPE group organisms (*Enterococcus faecium*, *Staphylococcus aureus*, *Klebsiella pneumoniae*, *Acinetobacter baumannii*, *Pseudomonas aeruginosa*, and *Enterobacter* spp.) are the most prevalent cause of multidrug resistant biofilm-associated chronic infections [5]. Certain biofilm producers, such as *Staphylococcus epidermidis* and the ESKAPE group, have been implicated in nosocomial infections from contaminated medical devices [6,7]. Biofilm plays a critical role in the pathogenesis of chronic diseases like tuberculosis and cystic fibrosis [8]. Treatment of biofilm-associated infections is becoming increasingly difficult due to multidrug resistance, and thereby they still pose a significant risk to human health. This study focuses on optimizing and evaluating different formulations to find effective therapeutics for biofilm-related bacterial infections.

Green tea, derived from *Camellia sinensis*, has held a significant place for a long time in traditional medicine. It is the second most popular beverage consumed in the world [9]. Epigallocatechin gallate (EGCG), the most abundant polyphenol/catechin in green tea, has been attributed numerous health benefits including antioxidant, anti-inflammatory, anti-cancerous, and antimicrobial properties [10,11,12,13]. United States Food and Drug Administration (FDA) classified EGCG as a safe compound due to its non-toxic nature and lack of side effects after application or consumption [14,15]. Many studies indicated antimicrobial [16,17] and anti-biofilm activity of EGCG on various Gram-positive and Gram-negative bacteria [18]. Parallel studies suggested synergism of antibiotics and EGCG on bacterial growth wherein EGCG is shown to enhance bacterial susceptibility, including methicillin-resistant *Staphylococcus aureus* (MRSA), *Porphyromonas gingivalis* (*P. gingivalis*) and *Klebsiella pneumoniae* (*K. pneumoniae*) to antibiotics [19,20,21,22,23].

Besides the beneficial properties, the natural water-soluble form of EGCG is relatively unstable, which affects its bioavailability and makes formulations difficult [24,25,26]. The rapid metabolism of EGCG results in a loss of therapeutic properties [19]. Several modifications of green tea polyphenols (GTPs) were synthesized to resolve the stability issue. Lipid-soluble analogs were found to be effective GTPs as they were highly stable with improved bioavailability [27]. Modified lipophilic polyphenols (LTPs) were shown to act synergistically with antibiotics to inhibit the growth of various Gram-positive and Gram-negative bacteria [19]. Another lipophilic EGCG derivative, EGCG-palmitate-based formulations exhibited sporicidal activity [28,29]. Recently, a derivative of EGCG known as epigallocatechin-3-gallate-stearate (EGCG-S) was synthesized by esterification of fatty acid making EGCG lipophilic and thereby, enhancing its absorption in the lipid bilayer. EGCG-S has been successfully shown to inhibit spores produced by *Bacillus* spp. and the growth of *Streptococcus mutans* (*S. mutans*) [30,31]. Additionally, EGCG-S was shown to improve the efficacy of antibiotics against various bacteria, thereby making it a potential synergistic anti-bacterial agent [32]. Various antibiotics have been previously shown to have synergism with EGCG/EGCG-S to inhibit bacterial growth [32]. The bacteria were more susceptible to specific antibiotics such as tetracycline and erythromycin respectively when used in combination with EGCG/EGCG-S [32].

In this study biofilm formation in five potentially pathogenic bacteria, including *Escherichia coli* (*E. coli*), *Mycobacterium smegmatis* (*M. smegmatis*), *Pseudomonas aeruginosa* (*P. aeruginosa*), *Staphylococcus aureus* (*S. aureus*), and *Staphylococcus epidermidis* (*S. epidermidis*) were studied. *E. coli* has been implicated in urinary tract infections (UTIs) [33]. The biofilm associated UTIs are frequently found in patients that use catheters [34,35]. The non-infectious *M. smegmatis* was used as a surrogate for *M. tuberculosis* as they share growth characteristics [36]. Both strains have been used as model organisms for biofilm studies [37]. Development of *M. tuberculosis* biofilm leads to cavity formation and necrosis in lung tissue [38]. *P. aeruginosa* is a major cause of nosocomial infections that fail to resolve with antibiotic treatment. Biofilms of these bacteria are found on implanted and indwelling devices [39]. *S. aureus* biofilm is one of the major hallmarks of cystic fibrosis respiratory infections. These infections have become refractory to antibiotics and have led to emergence of methicillin-resistant *S. aureus* (MRSA) [40]. *S. epidermidis*, a human commensal microorganism, is a causative agent of chronic infections in compromised hosts [41]. These infections are associated with the introduction of foreign biomaterials like catheters and prostheses [42]. All biofilm infections are becoming highly recalcitrant to host immune system and multiple drugs.

The classic biofilm measurements are based on direct cell enumeration by colony forming unit (CFU) counting and indirect biofilm accumulation by crystal violet (CV) staining. However, these techniques require biofilm resuspension and the potential carryover of the antimicrobials may skew the results [43]. Resazurin assay rapidly quantifies metabolic cell activity, is sensitive, simple, and requires no biofilm isolation [44]. This assay is a preferred choice for biofilm quantification [45] and is widely used in biofilm-associated studies [46,47,48].Therefore, this study utilized three independent quantitative assays, CFU analysis, CV staining, and resazurin, to ensure consistent data with high confidence. Furthermore, a fluorescent dye-based microscopic analysis was performed to study the viability of the cells. According to Clinical Laboratory Standards Institute (CLSI) Kirby-Bauer disk diffusion [49] profiling results, *E. coli* and *M. smegmatis* were intermediate to erythromycin while *P. aeruginosa* was resistant to erythromycin. Both *S. aureus* and *S. epidermidis* were intermediate to tetracycline. We previously reported that EGCG-S can enhance the erythromycin on Gram-negative bacteria such as *E. coli*, *P. aeruginosa* and converted the bacteria from antibiotic resistant or intermediate category to sensitive. EGCG-S can enhance tetracycline on *S. aureus* and *S. epidermidis* and converted the bacteria from antibiotic resistant or intermediate category to sensitive [32]. In this study, erythromycin was used on the Gram-negative and acid-fast bacteria; tetracycline was used on Gram-positive bacteria to study the synergistic action of EGCG-S and erythromycin/tetracycline in inhibiting biofilm production.

## 2. Results

### 2.1. The Effect of Erythromycin and EGCG-S on E. coli Biofilm Formation

The inhibitory effect of erythromycin (E) and EGCG-S (ES) treatments, individually and/or in combination, on *E. coli* biofilm formation was evaluated by CFU analysis. Based on the extent of inhibition, individual lethal dose (LD_50_) for erythromycin and EGCG-S were identified to be 15 μg/mL (E15) and 50 μg/mL (ES50) respectively. However, since biofilm is more tolerant to most of the treatment [1,2] it was hypothesized that the above-stated combinations will not be the optimal formulation for inhibiting biofilm production and thus, numerous combinations of erythromycin (E) and EGCG-S (ES) with variable concentrations were tested, along with individual treatments. The effect of individual and combination treatments on biofilm production in *E. coli* was monitored using colony-forming unit (CFU) assay. CFU measurements were used to calculate log reduction and percentage of inhibition. On testing the first combination comprising of individual LD_50_ concentrations (E15+ES50), the log reduction of 1.04 with only 94% inhibition was obtained. This data strongly suggests that combinations with higher concentrations are required to inhibit biofilm formation. In comparison to the control, the log reductions and percentage of inhibition showed by different formulations of E and ES are summarized in Table 1.

**Figure 1:**
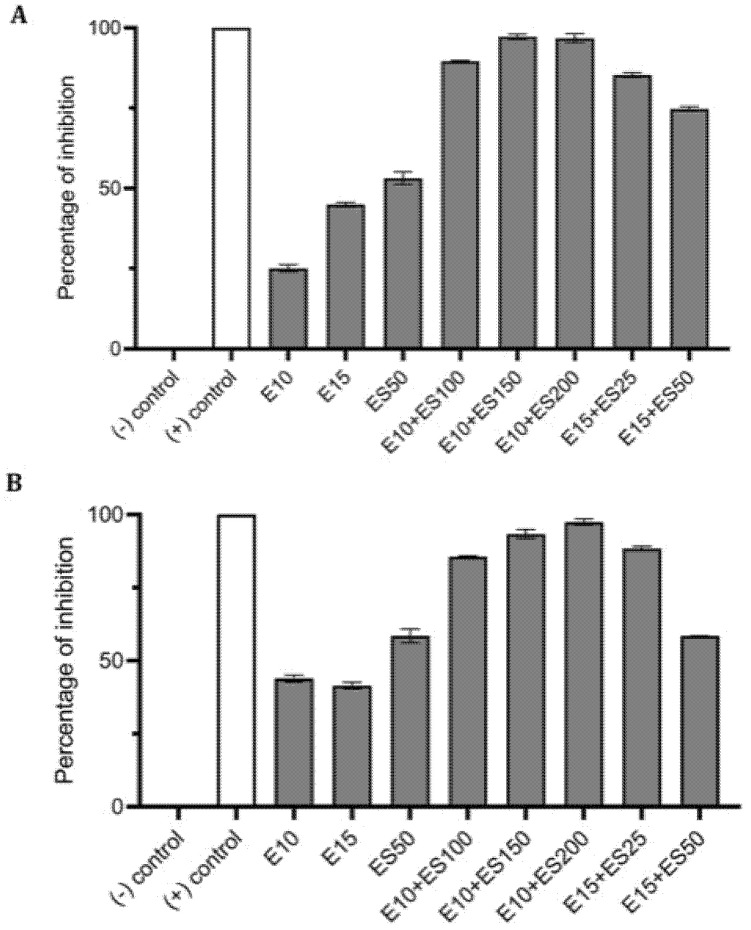
Effect of single and combined treatments of erythromycin (E) and EGCG-S (ES) on biofilm formation in *E. coli*. The percentage of inhibition for (**A**) crystal violet assay and (**B**) Resazurin assay were calculated from respective measurement. (-) control: phosphate buffer saline (PBS) buffer; (+) control: 10% bleach. Experiments were repeated in triplicates. Means are shown with SD. The percentage of inhibition with different concentrations of erythromycin and EGCG-S indicated that E10+ES200 inhibits *E. coli* biofilm formation most effectively.

**Figure 2:**
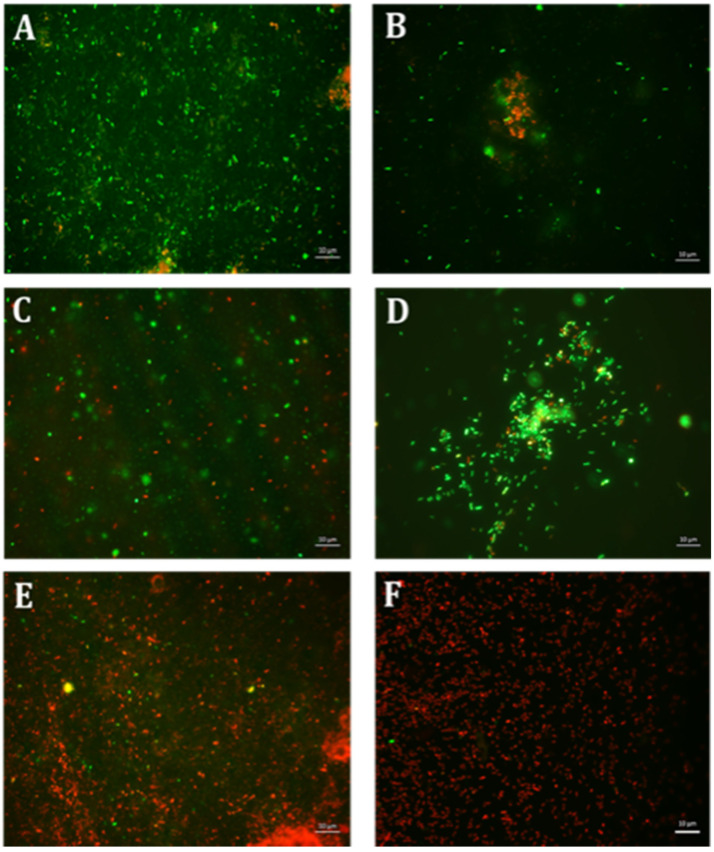
Fluorescence microscopic observation of cell viability on *E. coli* biofilm formation. E: erythromycin; ES: EGCG-S. Scale bar = 10 µm. (**A**) Control, (**B**) E10, (**C**) E15, (**D**) ES50, (**E**) E10+ES150, and (**F**) E10+ES200. The results indicated that both E10+ES150 and E10+ES200 severely affect the integrity of the cells and E10+ES200 is the most effective formulation.

Various formulations were tested for their inhibitory effect, and only two combinations E10+ES150 and E10+ES200 showed the highest log reduction of 1.3 and 1.22, respectively. The respective percentage of inhibition was 98% and 97% (*p* < 0.05). To further strengthen the CFU data, biofilm quantification was performed using CV and resazurin assay. The results of the CV analysis are represented in Figure 1A. Both E10+ES150 and E10+ES200 showed approximately 97% inhibition of biofilm using CV assay. However, CV assay is relatively error-prone, and thus fluorescence measurements using resazurin assay demonstrated that for samples treated with E10+ES150 and E10+ES200, the biofilm formation was significantly inhibited by 94% and 97% respectively, as depicted in Figure 1B. A highly significant difference compare with the negative control group was found (*p* < 0.05). Fluorescence microscopy was used to evaluate cell viability of treatment with E10, E15, ES50 and with two formulations, namely E10+ES150 and E10+ES200, as shown in Figure 2. The control/untreated group represented a bacterial cell population that fluoresced green (Figure 2A), indicating that most of the population was viable. Single treated with E10, E15, and ES50 did not affect the cell viability significantly. After treatment with 10 µg/mL of erythromycin and 200 µg/mL EGCG-S, almost the entire population fluoresced red indicating that most cells were dead (Figure 2F). Thus, the formulation containing E10+ES200 is significantly effective in inhibiting *E. coli* biofilm production.

### 2.2. The Effect of Erythromycin and EGCG-S on M. smegmatis Biofilm Formation

**Figure 3:**
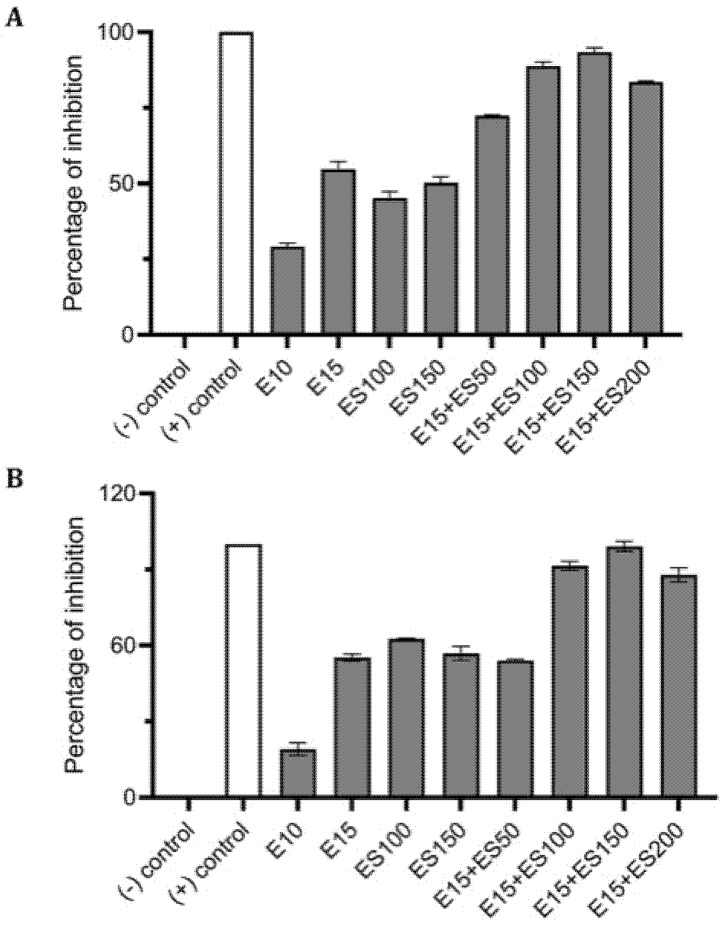
Effect of single and combinatorial treatments of erythromycin (E) and EGCG-S (ES) on biofilm formation in *M. smegmatis*. The percentage of inhibition for (**A**) crystal violet assay and (**B**) Resazurin assay were calculated from respective measurement. (-) control: PBS buffer; (+) control: 10% bleach. Experiments were repeated in triplicates. Means are shown with SD. The percentage of inhibition with different concentrations of erythromycin and EGCG-S indicated that E10+ES150 inhibits *M. smegmatis* biofilm formation most effectively.

**Figure 4:**
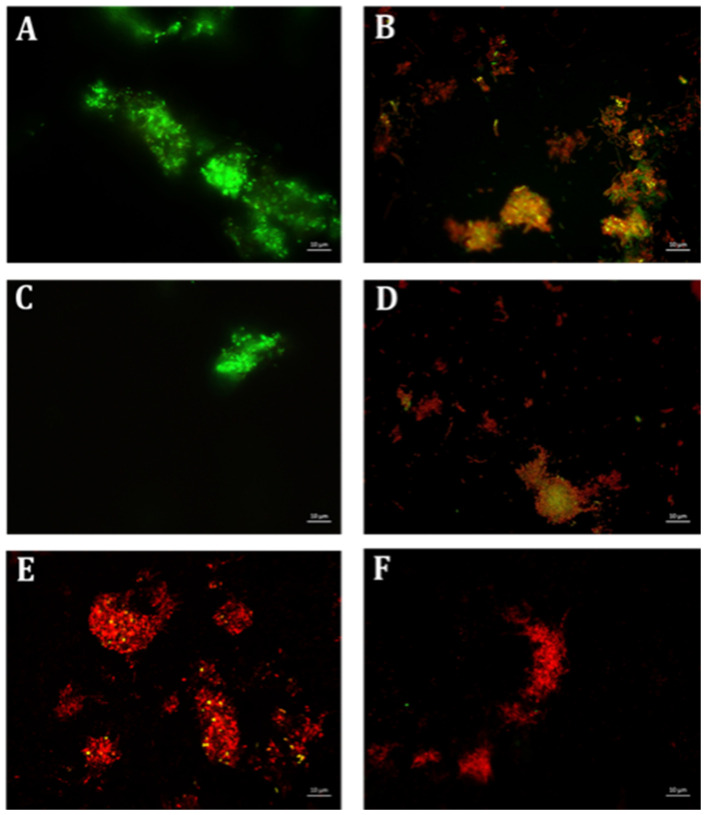
Fluorescence microscopic observation of cell viability on *M. smegmatis* biofilm formation. E: erythromycin; ES: EGCG-S. Scale bar = 10 µm. (**A**) Control, (**B**) E15, (**C**) ES100, (**D**) ES150, (**E**) E15+ES100, and (**F**) E15+ES150. The results indicated that E15+ES150 severely affect the viability of the cells.

Preliminary analysis identified 15 μg/mL of erythromycin (E15) and 100 μg/mL of EGCG-S (ES100) as LD_50_ for inhibition of bacterial biofilm formation. On comparing the inhibitory action on biofilm formation, based on CFU assay, the combination of E15+ES100 exhibited 72% inhibition with low log reduction, 0.51 (Table 2). Thus, varying the EGCG-S concentration in the combination, in CFU analysis, identified a formulation, E15+ES150, that significantly increased log reduction to 1.07 and showed 95% biofilm inhibition. Additionally, biofilm measurements, using CV and resazurin assay, calculated the percentage of inhibition of *M. smegmatis* biofilm to be 94% and 99%, respectively. The biofilm inhibition percentage for different treatments is shown in Figure 3. The percentage of inhibition was found to be statistically significant compared with the negative control at *p* < 0.05. The sample treated with a combination of E15+ES150 was further qualitatively tested for cell viability and was found to be non-viable, depicted in Figure 4, when compared to the control. Together, it can be concluded that the E15+ES150 formulation optimally inhibits the biofilm formation process in *M. smegmatis*.

### 2.3. The Effect of Erythromycin and EGCG-S on P. aeruginosa Biofilm Formation

**Figure 5:**
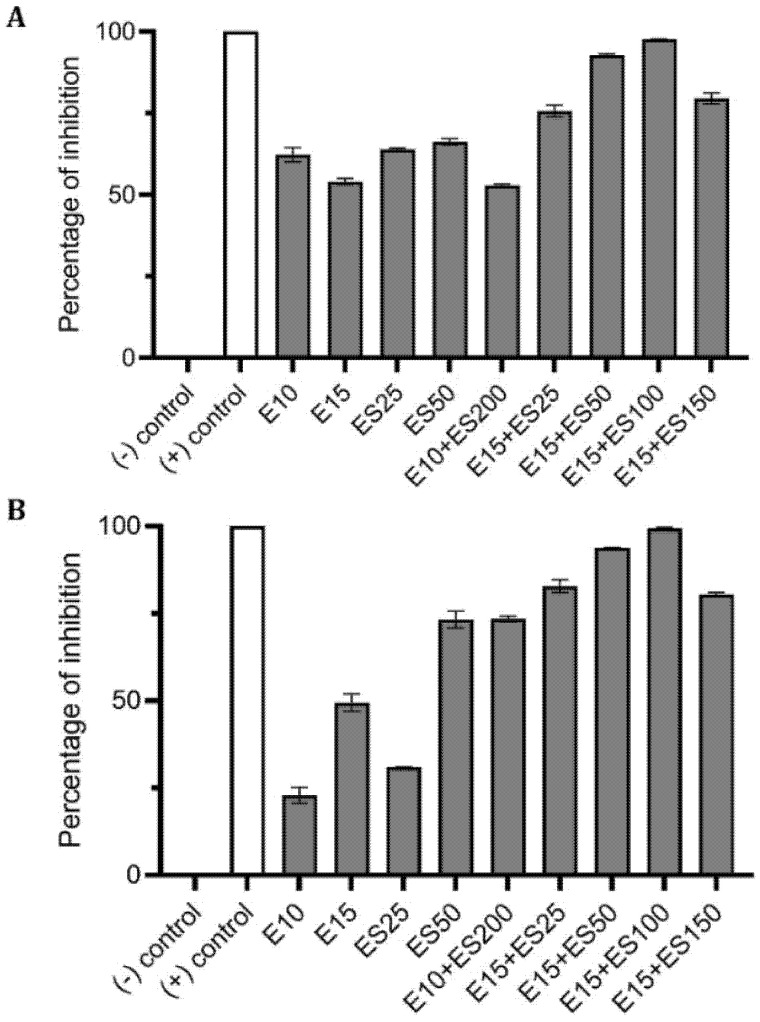
Effect of single and combinatorial treatments of erythromycin (E) and EGCG-S (ES) on biofilm formation in *P. aeruginosa*. The percentage of inhibition for (**A**) crystal violet assay and (**B**) Resazurin assay were calculated from respective measurement. (-) control: PBS buffer; (+) control: 10% bleach. Experiments were repeated in triplicates. Means are shown with SD. The percentage of inhibition with different concentrations of erythromycin and EGCG-S indicated that E10+ES100 concentration effectively inhibits biofilm formation of *P. aeruginosa*.

**Figure 6:**
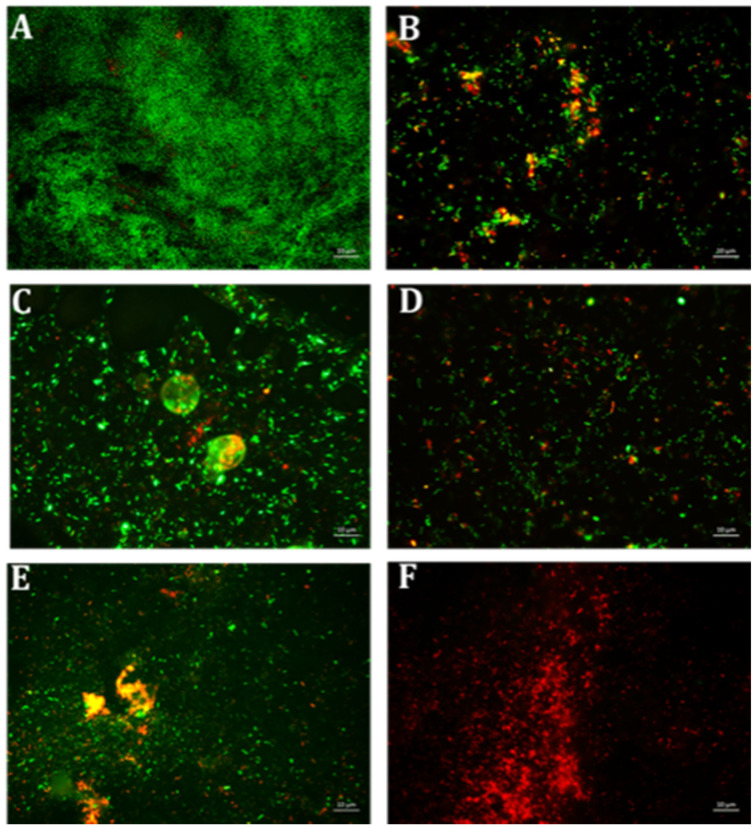
Fluorescence microscopic observation of cell viability on *P. aeruginosa* biofilm formation. E: erythromycin; ES: EGCG-S. Scale bar = 10 µm. (**A**) Control, (**B**) E15, (**C**) ES25, (**D**) ES50, (**E)** E15+ES50, and (**F**) E15+ES100. The results indicated that E15+ES100 severely affect the viability of the cells.

Biofilm formation of *P. aeruginosa* was evaluated in the presence of multiple concentrations of erythromycin and EGCG-S applied individually to determine E15 and ES50 as LD_50_. In addition, based on CFU assay (Table 3), a treatment combining the LD_50_ concentrations E15+ES50 demonstrated a mere 83% inhibition of biofilm production with only 0.61 log reduction of bacterial cells. Numerous combinations with higher concentrations of E and ES were further tested for their efficacy in inhibiting biofilm formation. Increased EGCG-S concentration to 100 µg/mL combined with E15 exhibited the highest log reduction of 0.88 with 95% inhibition of biofilm. Data obtained from CV and Resazurin assays, shown in Figure 5, identified E15+ES100 as optimal formulation as it inhibited biofilm formation by 97% and 99% respectively. The data within the groups was found to be highly significant (*p* < 0.05) compared with the negative control. Supplementary combinations of E15 with a low concentration of EGCG-S, ES50, gave a 91% inhibition of biofilm formation and hence, the optimal concentration was confirmed using qualitative bacterial viability assay. The microscopic images of control and treated samples are shown in Figure 6. The green fluorescence of the control sample indicated live bacterial cells in a biofilm. Mixed live and dead cells were observed in E15, ES25, ES50, E15+ES50 treated samples, while in treatment with the E15+ES100 formulation bacterial cells in the biofilm were no longer viable. Taken together, the biofilm formation process in *P. aeruginosa* is inhibited optimally by E15+ES100 formulation.

### 2.4. Synergistic Inhibitory Effect of Tetracycline and EGCG-S on Staphylococcus spp.

The *Staphylococcus* genus includes two important potential pathogenic strains: *S. aureus* and *S. epidermidis*. These Gram-positive organisms are well-known for chronic biofilm-associated infections. Individual and combination treatments were then applied to estimate the extent of inhibition on biofilm formation of *Staphylococcus* spp. using CFU assay; preliminary CFU analysis on biofilm formed by *S. aureus* identified 15 μg/mL of tetracycline (TE15) and 50 μg/mL EGCG-S (ES50) as LD_50_, respectively. The inhibition exhibited by a combination of TE15+ES50 on *S. aureus* biofilm was only 57% with a corresponding log reduction of 0.30. However, increasing the ES concentration led to conclude that TE15+ES200 formulation was optimal in reducing biofilm formation as it showed a log reduction of 0.75 and 94% inhibition (Table 4). For *S. epidermidis*, LD_50_ for inhibiting bacterial biofilm is 15 μg/mL of tetracycline (TE15) and 100 μg/mL EGCG-S (ES100) respectively. TE15+ES100 formulation comprising the LD_50_ concentrations that maximally inhibited *S. epidermidis* biofilm formation by 72% with a mere log reduction of 0.55 whereas other combinations with a higher concentration of ES increased the inhibitory effect. Finally, the TE15+ES250 formulation showed maximal inhibition of 95% and increased log reduction to 1.33 (Table 5).

**Figure 7:**
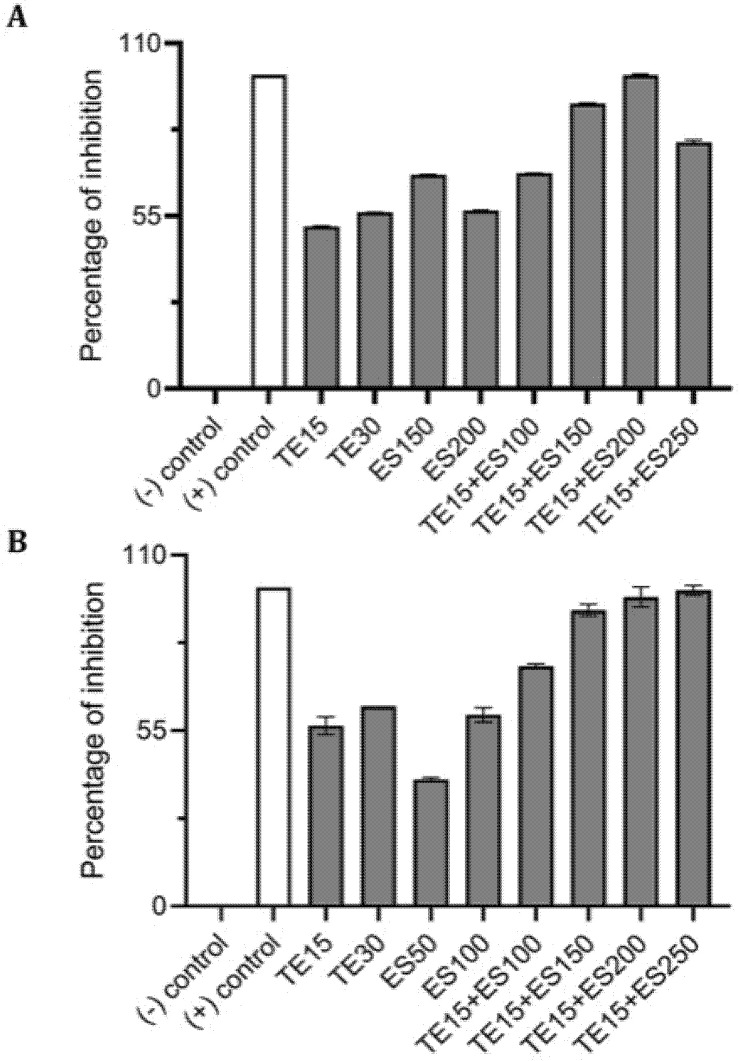
Effect of single and combinatorial treatments of tetracycline (TE) and EGCG-S (ES) on biofilm formation in *S. aureus*. The percentage of inhibition for (**A**) crystal violet assay and (**B**) Resazurin assay were calculated from respective measurement. (-) control: PBS buffer; (+) control: 10% bleach. Experiments were repeated in triplicates. Means are shown with SD. The percentage of inhibition with different concentrations of tetracycline and EGCG-S indicated that TE10+ES200 inhibits biofilm formation of *S. aureus* most effectively.

**Figure 8:**
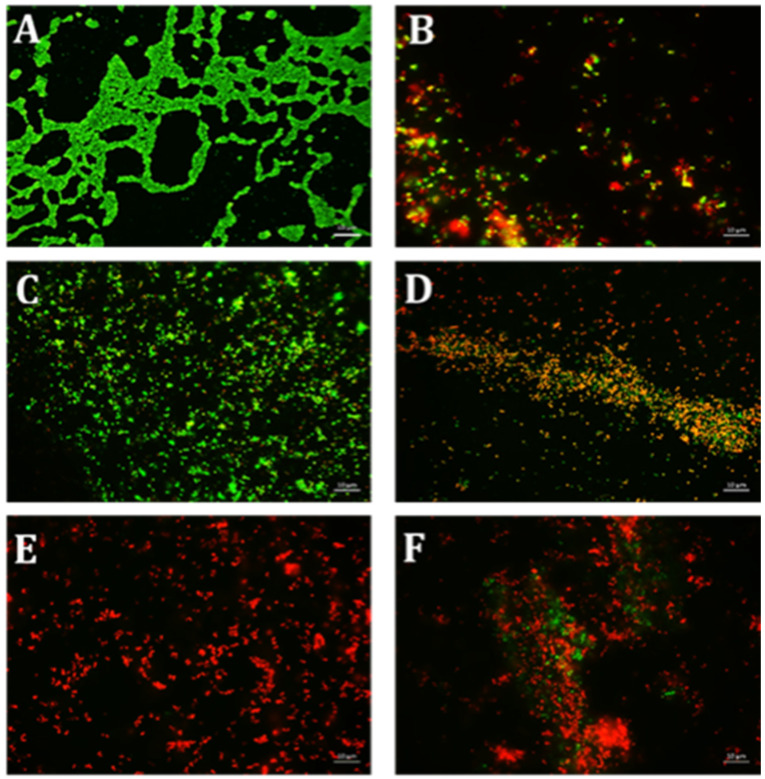
Fluorescence microscopic observation of cell viability on *S. aureus* biofilm formation. TE: tetracycline; ES: EGCG-S. Scale bar = 10 µm. (**A**) Control, (**B**) TE15, (**C**) ES100, (**D**) ES200, (**E**) TE15+ES200, and (**F**) TE15+ES250. The results indicated that TE15+ES200 severely affected the viability of the cells.

**Figure 9:**
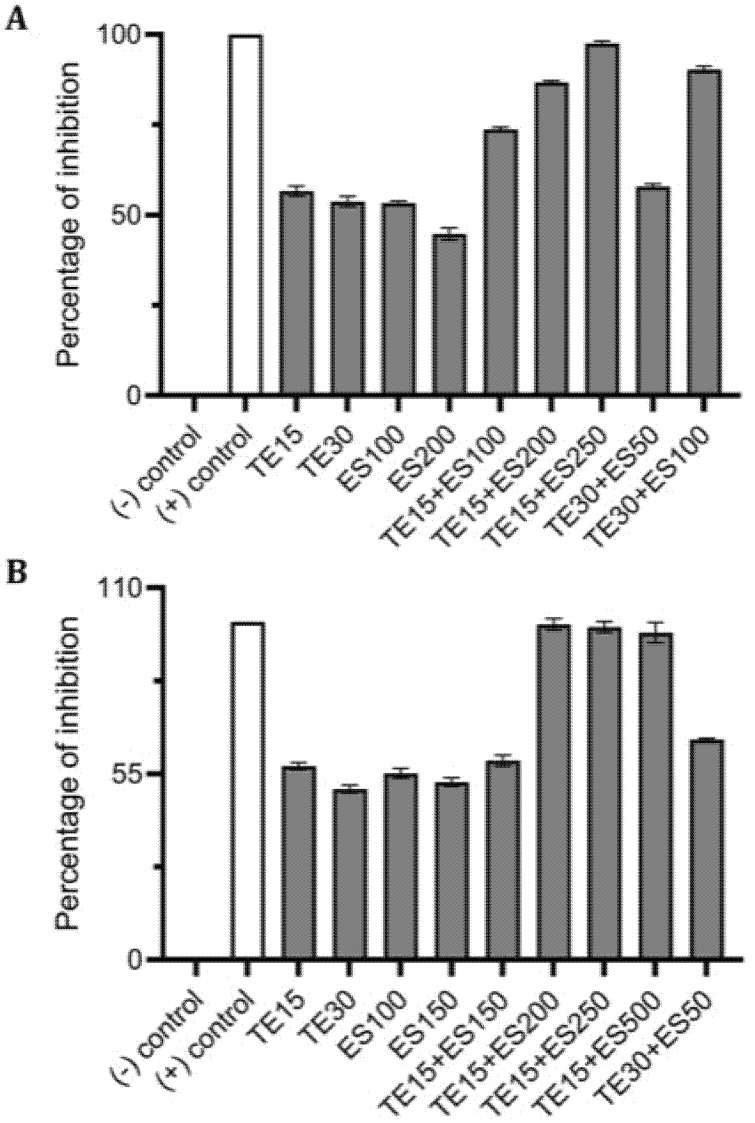
Effect of single and combined treatments of tetracycline (TE) and EGCG-S (ES) on *S. epidermidis* biofilm formation. The percentage of inhibition for (**A**) crystal violet assay and (**B**) Resazurin assay were calculated from respective measurement. (-) control: PBS buffer; (+) control: 10% bleach. Experiments were repeated in triplicates. Means are shown with SD. The percentage of inhibition with different concentrations of tetracycline and EGCG-S indicated that TE10+ES250 inhibits biofilm formation of *S. epidermidis* most effectively.

**Figure 10:**
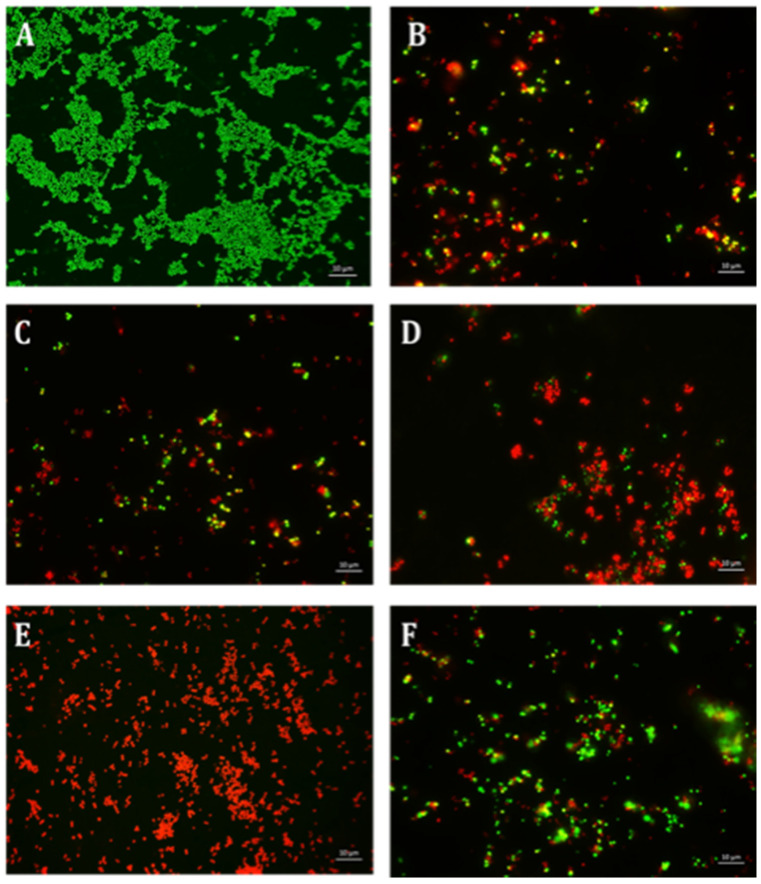
Fluorescence microscopic observation of cell viability on *S. epidermidis* biofilm formation. TE: tetracycline; ES: EGCG-S. Scale bar = 10 µm. (**A**) Control, (**B**) TE15, (**C**) ES100, (**D**) TE15+ES200, (**E**) TE15+ES250, and (**F**) TE30+ES100. The results indicated that TE15+ES250 severely affect the viability of the cells.

Using CV and resazurin assays to quantitively study biofilm formation in *S. aureus* identified that TE15+ES200 as the optimal combination exhibiting 97% and 98% inhibition of *S. aureus* biofilm respectively as shown in Figure 7. The TE15+ES200 formulation severely affected the viability of *S. aureus* cells (Figure 8). Similarly, in the case of *S. epidermidis* as shown in Figure 9, suggested that a combination of TE15+ES250 is effective in inhibiting biofilm formation to 99% and 97%, respectively. All quantitative data were subjected to statistical analyses and found that comparing with negative control was significant at *p* < 0.05. Furthermore, microscopic viability analysis demonstrated the death of *S. epidermidis* cells biofilm on treatment with above-stated combinations when compared to live cells in control, as shown in Figure 10. In conclusion, the optimal combination of green tea polyphenol, EGCG-S, and tetracycline that inhibited biofilm formation of *S. aureus* and *S. epidermidis* were found to be TE15+ES200 and TE15+ES250, respectively.

## 3. Discussion

EGCG exerts its antimicrobial effect on bacteria using various mechanisms, such as cell membrane damage, enzyme inhibition, impairment of fatty acid biosynthesis, and so forth [50]. EGCG has been shown to inhibit biofilm formation in diverse bacteria including *E. coli* [51], *P. aeruginosa* [23], *Staphylococcal* spp. [52], *S. mutans* [53], *P. gingivalis* [54], and *Fusobacterium nucleatum* [55] under in vitro and in vivo conditions. Recently, a detailed mechanism of action for EGCG was described using *E. coli* as a model system; EGCG synergistically targets amyloid curli fibers (anti-amyloidogenic) and cellulose synthesis, the two crucial processes of biofilm formation [56]. Furthermore, this study identified cell envelope stress as a second target for biofilm interference. Under stress, the cell downregulates the translation of trans-membrane proteins like protein complexes used in the biosynthesis of amyloid fibers and cellulose. This stress is potentially induced by the interaction of EGCG and the cell lipid bilayer [57], which can create temporal disturbances like perforations and grooves at the cell surface, thereby inducing cell damage [58,59,60]. EGCG impacts the integrity of the cell envelope, not only in *E. coli* but also in other bacteria, including *M. smegmatis* [61]. Although the trigger for this cell-envelope stress is unknown, it is proposed that reactive oxygen species (ROS) are involved in the bactericidal action of catechins [62]. ROS-mediated permanent damage has also been observed in a combination study of EGCG with antibiotic cefotaxime on *E. coli* cells [58]. In *Staphylococcus* spp. EGCG impairs the assembly of phenol-soluble modulins (PSMs) fibril formation and targets the preformed fibrils for disentanglement [63]. Another study found flavonoids can specifically prevent biofilm-associated proteins (Bap)–mediated biofilms [64]. Additionally, EGCG is shown to interfere with the polysaccharides that form the glycocalyx and bind peptidoglycan impairing the integrity of the cell wall, thereby suggesting that it can affect the initial attachment of biofilm to the surface [52]. EGCG has been found to be very effective in interfering with the action of amyloid fibers, FapC in *P. aeruginosa* [65] and amyloid proteins in *S. mutans* [66]. Besides, EGCG is involved in suppressing multiple virulence factors deployed by pathogenic bacteria to infections [67,68]. These studies suggest amyloid fibrils as a common target of EGCG, however EGCG can have multiple targets for a specific bacterial species and is efficient in interfering with multiple cellular processes without even entering a bacterial cell.

Some studies have suggested the synergism between EGCG and different antibiotics on inhibiting bacteria like methicillin-resistant *S. aureus* (MRSA), *P. gingivalis*, and *K. pneumoniae* [22,69,70], and on bacterial biofilms. Biofilm formation by pathogenic organisms has developed tolerance to elevated levels of antimicrobials [71]. Most studies demonstrate that EGCG can work synergistically with antibiotics by breaking down the extracellular matrix components, thereby favoring antibiotics penetration and action on bacterial cells in biofilms [21,22,23], while other studies report opposite results [67,72]. These counterproductive effects raise questions about the efficacy of EGCG as its stability and bioavailability fluctuate with research conditions. EGCG, a hydrophilic molecule with low membrane permeability and chemical stability [73], is not a suitable candidate to be formulated in therapeutic preparations without rapid oxidation and loss of antimicrobial activity. Thus, in this study, we have used a patented (US8076484B2) esterified derivative of EGCG, EGCG-Stearate (EGCG-S) which improves the bioavailability significantly [74,75]. Previously, we reported that EGCG-S can be used as an anti-spore agent, as it inhibits germination of spores produced by *Bacillus* species [30]. In 2018, our study highlighted the anti-cariogenic property of EGCG-S as it was able to inhibit the growth and biofilm formation of *S. mutans*, an etiological agent of dental caries [31]. Taken together, evidence shows EGCG-S inhibits spore germination and biofilm formation. However, whether the synergism of EGCG-S with antibiotics extends to interfere with biofilm formation is still unknown. To the best of our knowledge, this research is the first to illustrate that the combination of EGCG-S and antibiotics not only inhibits bacterial growth, but also biofilm formation.

## 4. Materials and Methods

### 4.1. Bacterial Cultures

Five potential pathogenic biofilm producers, *Escherichia coli* (ATCC^®^ CRM-8739), *Pseudomonas aeruginosa* (ATCC^®^ CRM-9027), *Staphylococcus aureus* (ATCC^®^ CRM-6538), *Staphylococcus epidermidis* (ATCC^®^ 14990), and *Mycobacterium smegmatis* (ATCC^®^ 19420) were grown aseptically on nutrient agar or broth. The stock cultures were stored at 4 °C. Fresh overnight cultures were maintained at 37 °C with constant shaking at 250 rpm. Gram staining was performed before each experiment to confirm the culture purity.

### 4.2. EGCG-S and Antibiotic Formulations

EGCG-S (US Patent 8076484) purchased from Camellix LLC (Evans, GA, USA), was dissolved in absolute ethanol to make a stock concentration of 10 mg/mL prior to formulations. The stock was diluted to the required concentrations for each experiment. Phosphate buffer saline (PBS) was used as a negative control, while 10% bleach was used as a positive control. Antibiotics erythromycin (E0774) and tetracycline (T3258), were purchased from Sigma Aldrich (St. Louis, MO, USA). The stock concentration (1000 μg/mL) was prepared by dissolving antibiotics in absolute ethanol. Final concentrations of ethanol in the working solutions were all less than 5%, which did not inhibit the growth of the bacteria. The solutions were filter sterilized and stored at −20 °C. The required concentrations were diluted from the stock aseptically prior to the experiment. The formulations consisted of varying concentrations of EGCG-S and antibiotics depending on the selected bacterium.

### 4.3. Quantitative Absorbance-Based Biofilm Measurement (Crystal Violet Assay)

The cultures were treated with EGCG-S and antibiotics alone and different formulations followed up by incubation at 37 °C for 4 days. The liquid was aspirated, the biofilm (if any) was washed and then stained with 0.1% crystal violet (CV) for 24 h. After strain aspiration and a final 1 × PBS wash, the biofilms were dried for 24 h. Biofilm was resuspended in 30% acetic acid and the optical density (OD) were recorded at 595 nm [76]. All experiments were performed in triplicate, with mean and standard deviation calculated. These readings were then used to determine the percentage of biofilm inhibition. 10% bleach and PBS were used as the positive and negative control, respectively.

Percentage of Inhibition = [(OD_Untreated_ − OD_Treated_)/OD_Untreated_] × 100 (1)

### 4.4. Quantitative Fluorescence-Based Biofilm Measurement (Resazurin Assay)

Fresh log-phase bacterial cultures were incubated at 37 °C for 4 days in 96-well plates. After the media was aspirated, the biofilm was rinsed with 1 × PBS and stained with a 200 μM Resazurin solution. After overnight incubation in the dark at 4 °C, fluorescence was measured at excitation and an emission wavelength of 560 nm and 590 nm respectively using a microplate reader (Infinite 200 PRO, Tecan, Männedorf, Switzerland) adjusted to the fluorescent mode [47]. The Relative Light Units (RLU) were measured, and the percentage (%) of inhibition was calculated for each treatment according to the following formula.

Percentage of Inhibition = [(RLU_Untreated_ − RLU_Treated_)/RLU_Untreated_] × 100 (2)

### 4.5. Quantitative Growth-Based Cell Viability Measurement (Colony Forming Unit Assay)

Bacterial cultures were incubated at 37 °C for 4 days in 24-well plates. The biofilm attached to the well plate was scrapped and suspended in 100 μL of deionized water. The samples were serially diluted (from 10^0^ to 10^−4^) and 100 μL of each dilution was spread plated on nutrient agar aseptically. The media plates were incubated overnight at 37 °C. Colony-forming units (CFUs) were recorded and percentage of inhibition was calculated using the following formula. LD_50_ represents the lethal concentration to 50% of the population.

Percentage of Inhibition = [(CFU_Untreated_ − CFU_Treated_)/CFU_Untreated_] × 100 (3)

Additionally, the CFU measurement was used to calculate log10 (fold) reduction using the following equation:

Log Reduction = Log_10_(CFU_Control_/CFU_Treated_) (4)

### 4.6. Qualitative Microscopy-Based Cell Viability Assay

The LIVE/DEAD^®^ BacLight™ Bacterial Viability Kit (Thermo Fisher, Waltham, MA, USA) was used according to the manufacture manual. The scrapped biofilms were grown in a 6-well plate with/without different treatments. After incubation at 37 °C for 4 days, the liquid was aspirated exposing the biofilms adhering to the well surface. The biofilms were then washed and stained with dye mixture. After proper staining coverslips were placed in the well to view under the microscope. The molecular probes used were SYTO 9, a green-fluorescent membrane-permeant dye that stains live cell, and propidium iodide (PI), red fluorescence dye that stains dead cells due to damaged cell membrane. All samples were viewed under a fluorescent microscope (Axio Scope A1, Carl Zeiss, München, Germany).

### 4.7. Statistical Analysis

All assays were performed in triplicate. Statistically significant differences between control and test were analyzed by one-way analysis of variance (one-way ANOVA) with Dunnett’s multiple comparison post-test at *p* < 0.05. The analyses were carried out in GraphPad Prism Software (GraphPad Software Inc., San Diego, CA, USA).

## 5. Conclusions

This study successfully demonstrated the synergistic inhibitory effects of a modified green tea polyphenol, EGCG-S, in combination with antibiotics on biofilm formation in five distinct potential pathogenic bacteria. Inhibition of biofilm formation was determined after an initial assessment of the inhibitory nature of EGCG-S and antibiotics on the growth of the cells. The LD_50_ of erythromycin for all Gram-negative bacteria (*E. coli*, *M. smegmatis*, and *P. aeruginosa*) is 15 µg/mL. The LD_50_ of EGCG-S is 50 µg/mL for *E. coli* and *P. aeruginosa*: 100 µg/mL for *M. smegmatis*. As for both Gram-positive bacteria (*S. aureus* and *S. epidermidis*), LD_50_ is 15 µg/mL for tetracycline and 50 µg/mL for EGCG-S. The optimal formulation of EGCG-S and antibiotics specific for every bacterium tested was determined by the combinatorial analysis of quantitative measurements from three different assays (CFU, CV, and resazurin). The colony-forming unit (CFU) assay provided preliminary information on the concentrations, of EGCG-S and antibiotics, necessary for their effects on biofilm formation. Although CFU data, indicating log-reduction of about 1.0, is not enough to strongly support synergism, it does reflect similar trends in comparison with the crystal violet (CV) and Resazurin assay. These experiments confirmed that EGCG-S in combination with antibiotics was able to maximally reduce biofilm formation in pathogenic bacteria. The findings were further confirmed by qualitative cell viability analysis and the optimal formulation for each bacterium was determined.

## Figures and Tables

**Table 1:** Colony-forming units (CFU/mL) and respective log reduction and percentage of inhibition for different treatments on biofilm formation in *E. coli*. E: erythromycin; ES: epigallocatechin-3-gallate-stearate (EGCG-S).

Treatments (μg/mL)	CFU/mL (Mean ± SD)	Log Reduction	Avg % Inhibition
E0+ES0	(1.81 ± 0.02) × 10^6^	0	0
E10	(1.34 ± 0.09) × 10^6^	0.13	27.01
E15	(1.10 ± 0.03) × 10^6^	0.22	40.52
ES50	(8.72 ± 0.90) × 10^5^	0.32	53.39
E10+ES100	(3.00 ± 0.23) × 10^5^	0.78	86.07
E10+ES150	(9.00 ± 0.43) × 10^4^	1.30	98.04
E10+ES200	(1.10 ± 0.15) × 10^5^	1.22	96.93
E15+ES25	(1.85 ± 0.10) × 10^5^	0.99	92.65
E15+ES50	(1.65 ± 0.07) × 10^5^	1.04	93.77

**Table 2:** Colony-forming units (CFU/mL) and respective log reduction and percentage of inhibition for different treatments on biofilm formation in *M. smegmatis*. E: erythromycin; ES: EGCG-S.

Treatments (μg/mL)	CFU/mL (Mean ± SD)	Log Reduction	Avg % Inhibition
E0+ES0	(1.60 ± 0.07) × 10^5^	0	0
E10	(1.18 ± 0.05) × 10^5^	0.13	27.16
E15	(8.30 ± 0.65) × 10^4^	0.29	49.91
ES100	(7.75 ± 0.62) × 10^4^	0.31	53.33
ES150	(9.20 ± 0.25) × 10^4^	0.24	43.99
E15+ES50	(4.40 ± 0.33) × 10^4^	0.56	75.20
E15+ES100	(4.85 ± 0.23) × 10^4^	0.52	72.33
E15+ES150	(1.35 ± 0.03) × 10^4^	1.07	94.84
E15+ES200	(3.75 ± 0.11) × 10^4^	0.63	79.38

**Table 3:** Colony-forming units (CFU/mL) and respective log reduction and percentage of inhibition for different treatments on biofilm formation in *P. aeruginosa*. E: erythromycin; ES: EGCG-S.

Treatments (μg/mL)	CFU/mL (Mean ± SD)	Log Reduction	Avg % Inhibition
E0+ES0	(3.40 ± 0.36) × 10^6^	0	0
E10	(1.41 ± 0.09) × 10^6^	0.38	61.14
E15	(1.53 ± 0.12) × 10^6^	0.35	57.47
ES25	(2.00 ± 0.12) × 10^6^	0.23	42.99
ES50	(1.74 ± 0.14) × 10^6^	0.29	51.10
E10+ES200	(1.39 ± 0.05) × 10^6^	0.39	64.65
E15+ES25	(1.48 ± 0.15) × 10^5^	0.36	59.12
E15+ES50	(8.25 ± 0.85) × 10^5^	0.61	83.00
E15+ES100	(4.45 ± 0.36) × 10^5^	0.88	95.10
E15+ES150	(1.44 ± 0.07) × 10^6^	0.37	63.02

**Table 4:** Colony-forming units (CFU/mL) and respective log reduction and percentage of inhibition for different treatments on biofilm formation in *S. aureus*. E: erythromycin; ES: EGCG-S.

Treatments (μg/mL)	CFU/mL (Mean ± SD)	Log Reduction	Avg % Inhibition
TE0+ES0	(4.41 ± 0.34) × 10^6^	0	0
TE15	(2.29 ± 0.18) × 10^6^	0.29	52.65
TE30	(2.00 ± 0.16) × 10^6^	0.34	59.38
ES50	(3.18 ± 0.27) × 10^6^	0.14	30.14
ES150	(1.59 ± 0.11) × 10^6^	0.44	69.40
ES200	(2.01 ± 0.15) × 10^6^	0.34	59.07
TE15+ES50	(2.20 ± 0.34) × 10^6^	0.30	57.37
TE15+ES100	(2.03 ± 0.20) × 10^6^	0.34	61.73
TE15+ES150	(9.45 ± 0.37) × 10^5^	0.67	89.88
TE15+ES200	(7.75 ± 0.62) × 10^5^	0.75	94.35
TE15+ES250	(1.58 ± 0.04) × 10^6^	0.45	73.53

**Table 5:** Colony-forming units (CFU/mL) and respective log reduction and percentage of inhibition for different treatments on biofilm formation in *S. epidermidis*. TE: tetracycline; ES: EGCG-S.

Treatments (μg/mL)	CFU/mL (Mean ± SD)	Log Reduction	Avg % Inhibition
TE0+ES0	(4.72 ± 0.15) × 10^6^	0	0
TE15	(2.10 ± 0.35) × 10^6^	0.35	55.52
TE30	(2.03 ± 0.16) × 10^6^	0.37	56.95
ES50	(3.15 ± 0.29) × 10^6^	0.18	33.36
ES100	(2.35 ± 0.50) × 10^6^	0.30	50.20
TE15+ES100	(1.34 ± 0.23) × 10^6^	0.55	71.69
TE15+ES200	(4.78 ± 0.64) × 10^5^	0.99	89.87
TE15+ES250	(2.19 ± 0.25) × 10^5^	1.33	95.36
TE15+ES500	(1.71 ± 0.01) × 10^6^	0.44	63.72

## Data Availability

Data is contained within the article.
